# Using Data to Improve Programs: Assessment of a Data Quality and Use Intervention Package for Integrated Community Case Management in Malawi

**DOI:** 10.9745/GHSP-D-17-00103

**Published:** 2017-09-27

**Authors:** Elizabeth Hazel, Emmanuel Chimbalanga, Tiyese Chimuna, Humphreys Nsona, Angella Mtimuni, Ernest Kaludzu, Kate Gilroy, Tanya Guenther

**Affiliations:** a Johns Hopkins Bloomberg School of Public Health, Baltimore, MD, USA.; b Save the Children, Lilongwe, Malawi.; c Independent Consultant, Lilongwe, Malawi.; d Ministry of Health, Lilongwe, Malawi.; e Formerly with the Ministry of Health, Lilongwe, Malawi.; f Maternal and Child Survival Program, Washington, DC, USA.; g Save the Children, Washington, DC, USA.

## Abstract

Health Surveillance Assistants (HSAs) have been providing integrated community case management (iCCM) for sick children in Malawi since 2008. HSAs report monthly iCCM program data but, at the time of this study, little of it was being used for service improvement. Additionally, HSAs and facility health workers did not have the tools to compile and visualize the data they collected to make evidence-based program decisions. From 2012 to 2013, we worked with Ministry of Health staff and partners to develop and pilot a program in Dowa and Kasungu districts to improve data quality and use at the health worker level. We developed and distributed wall chart templates to display and visualize data, provided training to 426 HSAs and supervisors on data analysis using the templates, and engaged health workers in program improvement plans as part of a data quality and use (DQU) package. We assessed the package through baseline and endline surveys of the HSAs and facility and district staff in the study areas, focusing specifically on availability of reporting forms, completeness of the forms, and consistency of the data between different levels of the health system as measured through results verification ratio (RVR). We found evidence of significant improvements in reporting consistency for suspected pneumonia illness (from overreporting cases at baseline [RVR=0.82] to no reporting inconsistency at endline [RVR=1.0]; *P*=.02). Other non-significant improvements were measured for fever illness and gender of the patient. Use of the data-display wall charts was high; almost all HSAs and three-fourths of the health facilities had completed all months since January 2013. Some participants reported the wall charts helped them use data for program improvement, such as to inform community health education activities and to better track stock-outs. Since this study, the DQU package has been scaled up in Malawi and expanded to 2 other countries. Unfortunately, without the sustained support and supervision provided in this project, use of the tools in the Malawi scale-up is lower than during the pilot period. Nevertheless, this pilot project shows community and facility health workers can use data to improve programs at the local level given the opportunity to access and visualize the data along with supervision support.

## Abstract (precis)

Use of simple wall charts by community and facility health workers to collect and visualize data helped inform data-based decision making for community health education activities, tracking stock-outs, staffing decisions, and other programming issues. Since intervention scale-up, however, use of the wall chart has dropped, demonstrating need for continued investment in supportive supervision.

## BACKGROUND

Integrated community case management (iCCM), in which community health workers (CHWs) are trained to assess, classify, and treat diarrhea, malaria, and pneumonia in children under 5 years of age, is a globally recognized strategy to reduce childhood illness in high-burden countries.^1^ National iCCM programs have been implemented widely around the world; by 2013, 28 of 42 high-burden countries in sub-Saharan Africa were implementing iCCM for malaria, pneumonia, and diarrhea. ^2^ Various studies have shown that while program strength varies across settings, iCCM programs are capable of providing high-quality care.^3^^–^^5^

Malawi was one of the first sub-Saharan African countries to implement iCCM. In 2008, the Integrated Management of Childhood Illness (IMCI) unit of the Ministry of Health (MOH) introduced iCCM in approximately 3,500 rural hard-to-reach communities, located 8 or more kilometers from health facilities.^6^ iCCM is provided by Health Surveillance Assistants (HSAs), a paid government cadre who serve catchment areas of approximately 2,000 population. All HSAs have a minimum of a 10th-grade education and complete a 10-week training program on preventive and promotive health care.^6^ Those selected for iCCM complete an additional 6-day training course using training materials adapted from guidelines from the World Health Organization (WHO) and the United Nations Children's Fund (UNICEF).^6^ During the initial scale-up of iCCM, major implementing partners included WHO, UNICEF, and the United Nations Population Fund (UNFPA) jointly; Save the Children; Population Services International; and Concern International. ^7^

A strong health information system is one of the building blocks of a functional health system.^8^ Program managers and MOH staff require feasible, timely, reliable, and valid measures of implementation to identify problems, begin quality improvement processes, and determine progress. Among many implementation challenges, country programs have struggled to measure and monitor implementation and overall progress in iCCM. In particular, collecting data on a routine basis from dispersed and hard-to-reach CHWs is a major challenge. Even facility-based routine data on program implementation are often incomplete and of questionable quality, and the collected data are not easily accessible for use in analysis and interpretation.^9^^–^^11^ Health worker training on data use is limited at all levels of the health system with weak linkages to the decision-making process for program improvement.^12^

A strong health information system is one of the building blocks of a functional health system.

At the time of this study, Malawi had a mature national iCCM program (with heavy partner support) and a functioning monitoring system in which iCCM data were integrated with the health management information system (HMIS). A lot of data was being generated but little of it was being analyzed and used for program improvement. Reporting completion and quality varied; districts with partner support tended to have greater reporting than other districts due to outreach activities for poor-reporting village clinics. The data were being compiled but not used at the health facility or HSA levels, and national-level data were aggregated and reported at the district level.

At the time of this study, iCCM data were being compiled but not used at the health facility or HSA levels.

We worked with district health staff and partners to develop and pilot a program to improve data interpretation and use at the health worker level. The objective of the data quality and use (DQU) package was to provide HSAs and health facility and district staff training and tools to analyze and interpret iCCM monitoring data, with the overall goal of improving data quality and empowering health workers to make timely data-based decisions to improve programs. In this article, we describe and evaluate implementation of the DQU package in 2 districts of Malawi.

## METHODS

### Description of the iCCM Monitoring and Evaluation System

**FIGURE 1:**
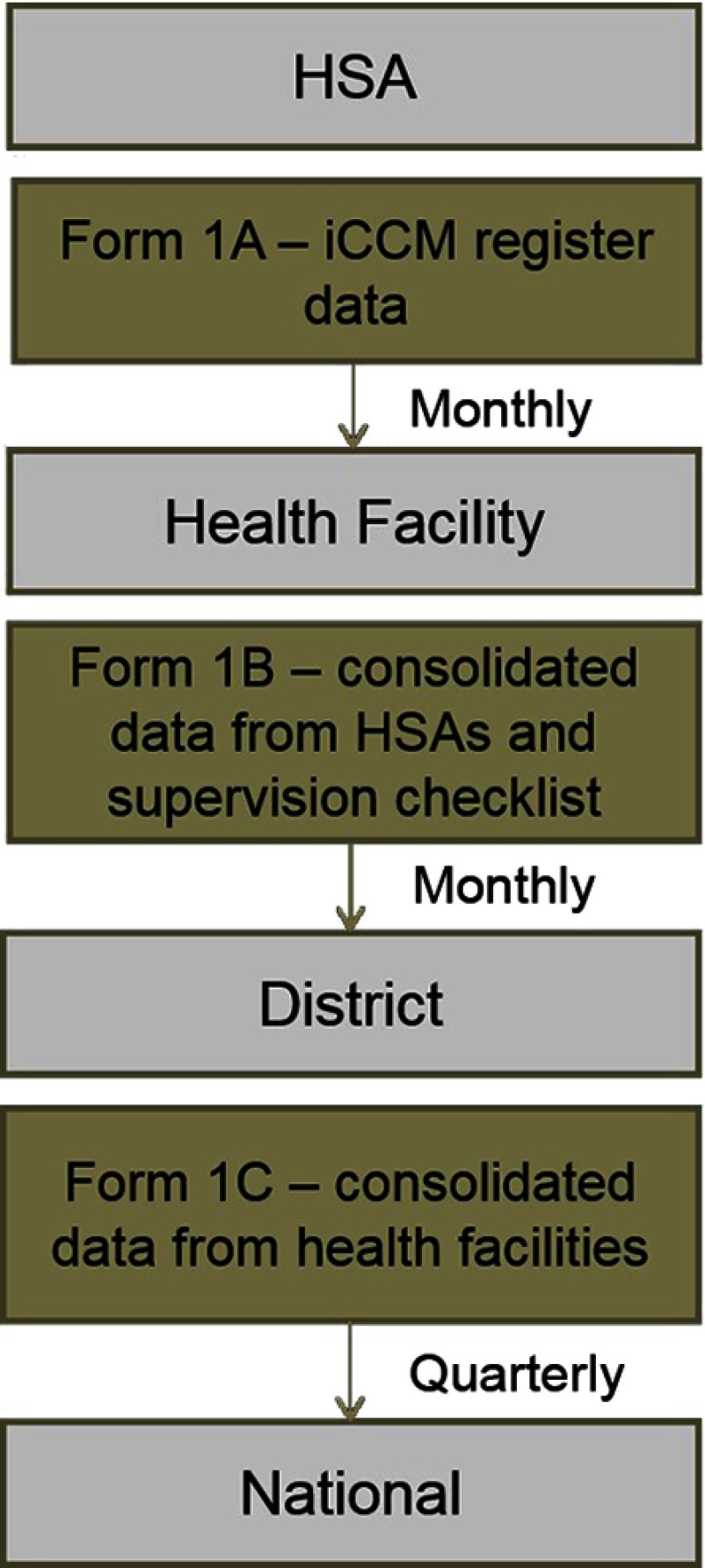
Malawi iCCM Routine Reporting Data Flow Abbreviations: HSA, Health Surveillance Assistant; iCCM, integrated community case management.

In 2011, the Malawi MOH and partners developed a set of 11 indicators to routinely measure implementation strength of iCCM, covering areas of HSA training, deployment and availability, supervision, supply chain management, and service delivery (Box 1). The iCCM patient registers and reporting forms were updated to reflect these indicators, and district rollout of the revised tools started in November 2011, with a target of reaching 3 new districts per quarter. Figure 1 shows the routine reporting structure and specifies the associated iCCM tools in place during the study period (2012–2013). Each HSA completed Form 1A summarizing data from its iCCM register on cases seen by type of condition, referrals, supplies dispersed, supervision received, and any child deaths in their catchment area. Health facility staff consolidated the data from HSAs and their supervision and mentoring checklists into Form 1B and submitted the form to the district IMCI coordinator. The IMCI coordinator consolidated the data using Form 1C on a quarterly basis.

BOX 1 **National iCCM Monitoring Indicators Used in Malawi**

1. Number of HSAs trained
2. Number of HSAs deployed
3. Number of “hard-to-reach” areas with a trained HSA
4. Number of iCCM-trained CHWs who have seen a sick child in the past 7 days
5. Number of iCCM-trained HSAs with no stock-outs of greater than 7 days of key medicines within the last 3 months (antibiotic, antimalarial, oral rehydration salts [ORS], zinc)
6. Number of iCCM-trained HSAs with no stock-outs of lifesaving medicines within the last 3 months (antibiotic, antimalarial, ORS)
7. Number of iCCM-trained HSAs supervised in iCCM in the last 3 months
8. Number of iCCM-trained HSAs supervised in iCCM in the last 3 months with reinforcement of clinical practice (case observation, case scenarios, mentoring at health facility)
9. Number of iCCM-trained HSAs residing in their catchment area
10. Number of sick children assessed each month by major condition
11. Number of sick children treated each month by major condition

### DQU Implementation

We purposefully selected 2 study districts, Dowa and Kasungu, using the following criteria: presence of statistical clerks at the health facility level; close proximity to the capital city to reduce transport costs; implementation of the revised iCCM reporting forms and data for at least 1 month available; and differing partner support. In Dowa, implementation of iCCM was initiated in 2010 with support from Save the Children, and by 2012, 224 HSAs had been trained and deployed to hard-to-reach areas. In Kasungu, iCCM implementation began in 2009 with support from WHO and UNICEF, and 198 HSAs were trained and deployed by 2012.

In 2013, Nutley and Reynolds provided a logical framework for how interventions can increase data use and demand.^13^ Table 1 presents an overview of the DQU package according to this framework. The DQU package was developed through national- and district-level consultations with the MOH and informed by a baseline assessment of data quality (see next section for details). It was implemented at the health facility and HSA levels. We developed data analysis and interpretation training guidelines based on resources from MEASURE Evaluation.^14^ We also developed wall chart templates to display iCCM implementation strength data at the health facility and HSA levels (Supplement 1 and Supplement 2).

**TABLE 1.** Data Quality and Use Package Implemented in Malawi

Processes to Improve Data Demand and Use	Elements of the DQU Package Design and Implementation
1. Assess and improve the data use context	Participatory baseline data quality assessment, using tools based on the PRISM framework and MEASURE Evaluation data quality audit tools, and involving national and district iCCM managers
2. Engage data users and data producers	Engagement of HSAs, health facility staff, district manager, and national IMCI unit staff in designing the package training materials and tools
3. Improve data quality *(quality defined as accurate, complete, and timely)*	Baseline data quality assessment to identify barriers to data quality Provision of calculators to HSAs to improve accuracy of monthly tallies Refresher training on how to complete routine registers and reports
4. Improve data availability *(availability defined as data synthesis, communication, and access to data)*	Development and dissemination of standardized wall charts to display data onsite Training on analysis, interpretation, and presentation of data for HSAs, health facility, and district staff
5. Identify information needs	Consultations with national, district, and facility staff and HSAs to document and prioritize information needs for monitoring iCCM Working with district IMCI coordinators to identify reporting “benchmarks” and “action thresholds” and to agree on response to levels below the agreed-upon action threshold
6. Build capacity in data use core competencies	General training on data management, use, and interpretation Involvement of district staff in data collection and supervision to build leadership capacity and to better advocate for data use in their districts
7. Strengthen the organization's data demand and use infrastructure	Development of written guidance on iCCM data analysis and use Provision of data display templates
8. Monitor, evaluate, and communicate results of data use interventions	Evaluation of the DQU package through mixed-methods, pre-post assessment and estimation of cost for scale-up Dissemination of findings in Malawi and globally to leverage resources to expand to other districts (and countries)

Abbreviations: DQU, data quality and use; HSA, Health Surveillance Assistant; iCCM, integrated community case management; IMCI, Integrated Management of Childhood Illness.

Based on the Nutley and Reynolds logic model to strengthen data demand and use.^13^

We developed wall chart templates to display iCCM data at the health facility and HSA levels.

In December 2012, we conducted a 2-day training of trainers (TOT) with 17 district staff including IMCI program coordinators, deputy coordinators, pharmacy technicians, and HMIS officers. The participants reviewed the materials, provided feedback, and were equipped to provide training to the HSA supervisors and HSAs through demonstration and mock/practice trainings. We gave them an additional 2-day refresher training in February 2013 prior to the package implementation. Two Save the Children staff facilitated the training with support from the MOH.

Beginning in February 2013, all senior HSAs (or other HSA supervisors) and HSAs implementing iCCM (N=426) were trained through 69 health facilities. Each training session at a facility took one-half day and the trainers were able to cover 2 facilities per day. The total training time per district was 2 weeks maximum. All participants convened at the catchment health facility for the training. District staff, periodically supervised by study staff, conducted the trainings, which covered refreshers on completing the monthly reporting form and completing the wall charts with instruction, demonstration, and practice. At the conclusion of the training, participants were instructed to complete the wall charts beginning (retrospectively) in January 2013. Due to a funding interruption, participants in Kasungu district were not trained until April 2013 (while those in Dowa district were trained in February 2013).

Implementation of the DQU package was designed to be flexible to the needs and context of each health facility to improve uptake and sustainability. District IMCI coordinators and health facility staff were encouraged to determine many of the implementation details, such as how the wall charts were filled in and where to display them. External supervision of the package implementation was minimal, as we aimed to assess effectiveness in a “real-world” scenario and determine feasibility for further scale-up. A 1-week supervision field mission to Kasungu district was conducted by study staff to observe template use in health facilities and village clinics. District staff conducted supervisory and mentoring visits as part of their routine supervision activities to HSAs and senior HSAs. At the time of this study, approximately one-third of the HSAs were receiving quarterly supervision with reinforcement of clinical practices.^15^ The package was implemented in Kasungu from April 2013, and in Dowa from February 2013, until the endline DQA in July 2013, for a minimum of 3 months of implementation.

### DQU Evaluation

We conducted a pre- and post-process evaluation to determine any changes in reporting availability, completeness, and consistency. We also documented use of the wall charts and specific program decisions resulting from the chart data.

#### Data Collection

We conducted 2 data quality assessments (DQAs), a baseline in 2012 and an endline in 2013. We used a mixed-method tool that included iCCM register reviews and a structured interview guide with open-ended responses. The tools were adapted from the frameworks and assessment tools for data quality audits and for assessing the Performance of Routine Information Systems Management (PRISM).^16^^–^^18^ This framework and these tools have been used in many settings to measure the strength of routine reporting in health systems. We used 3 data collection forms, one for each level of the health system—the HSA level (Form 1), facility level (Form 2), and district level (Form 3). The forms were in English and the interviews were in Chichewa. The open-ended qualitative interview responses were summarized and recorded in English by the interviewers. All interviewers were fluent in English and Chichewa.

In each district, we selected the district hospital and randomly selected 4 health facilities. The district hospital was selected because it has the largest patient load and the teams already had to travel there for the district coordinator interviews. We interviewed the senior HSAs at the 5 facilities (10 total selected) and 4 HSAs from each selected health facility. The HSAs were randomly selected from a full list of HSAs trained and deployed in iCCM in the hard-to-reach areas obtained from the MOH/IMCI unit and partners. Kasungu and Dowa district health staff were invited to work as data collectors during this exercise to provide valuable input during the study, collecting data from each other's districts to minimize bias.

The tools and protocol for the endline DQA were the same as the baseline DQA. We revisited the previously selected 10 health facilities and HSAs in the catchment area. Any HSA not available during the period of data collection was replaced with a neighboring HSA. Baseline data collection tools were updated to include reviews of the existing data collection and compilation tools and to capture perceptions and use of the DQU package.

Baseline data collection was carried out over a 2-week period in June 2012, and endline data collection during a 2-week period in July 2013. All reviews of data and tools pertained to the previous 2 completed months. Teams visited the district offices and health facilities to apply data collection Form 1 and Form 2. Form 1 collected information for all the health facilities reporting to the district (not just the 10 selected) and Form 2 collected information for all the iCCM-trained HSAs reporting to the 10 health facilities (not just the 40 selected).

The selected HSAs were asked to convene at their health facility and to bring their iCCM register for review; the data collection team used DQA Form 3 for interviews and register reviews. Two senior staff, from the study office and the MOH, attended the interviews in both districts to provide consistency and supervision. Data were collected on paper and electronically scanned for analysis. Interviews were conducted at the selected health facilities.

Finally, to inform further scale-up, we estimated the cost of the intervention by tracking expenditures for key package inputs such as printing, trainings, and supervision.

#### Data Analysis

Quantitative data entry and cleaning were conducted using Microsoft Excel 2010. STATA 11 was used for the analysis.^19^ Single data entry was used and all data in the final analysis were cross-checked against the hard copies. We assessed changes in:

1. Availability of forms submitted for the previous month
2. Completeness of submitted forms
3. Consistency, measured through results verification ratio (RVR)^17^

The RVR indicates the level of consistency of routine reporting systems. In other words, at the HSA level, we compared the number of cases entered in the iCCM registers to what was entered on the reporting form for that month by the HSA and submitted to the facility. At the health facility level, we compared the number of cases from the monthly reporting forms submitted by all the HSAs reporting to that facility with what the facility reported to the district for that particular month. The RVR was calculated for the number of diarrhea, fast-breathing (suggesting pneumonia), and fever illness cases; male and female cases; and number of cotrimoxazole, oral rehydration salts, and lumefantrine-artemether dispensed. We considered an RVR of less than 0.8 (20% overreporting) or more than 1.2 (20% underreporting) to be problematic.

We described changes in the availability and completeness of the routine forms for the previous reporting month: May 2012 for the baseline and June 2013 for the endline. For reporting consistency, we used the most recent month with a form submitted: April or May 2012 and May or June 2013. We used a paired *t* test analysis, taking into account the clustering of HSAs at their catchment facilities, to examine whether the RVRs changed from baseline to endline. To evaluate data display and use, we determined the proportion of HSAs and health facility staff using and displaying the wall charts and the proportion with completed wall charts for the study period.

For the qualitative data, narrative summary was used to describe reported successes, challenges, and the perceived value of the DQU package. Selected quotes are presented in this article that describe how the data were used to improve programs.

The cost of implementing the package was abstracted from Save the Children expenditure data related to the project. We calculated the cost by dividing the total cost of training, printing, and supervision by the number of facilities in both districts to generate an estimated cost per health facility.

### Ethical Review

The study was submitted to the Johns Hopkins Bloomberg School of Public Health Institutional Review Board and was deemed as non-human subjects research and exempt from review. We obtained permission from the district health offices before beginning implementation and data collection.

## RESULTS

For the 2012 baseline DQA, we interviewed 10 senior HSAs in the 10 selected facilities and 38 iCCM-trained HSAs. One HSA was unavailable for interview and 1 selected health facility only had 3 HSAs trained in iCCM. For the 2013 follow-up DQA, we completed interviews with 9 senior HSAs and 36 HSAs. We were unable to conduct interviews at Dowa hospital. The majority of interviewees in the follow-up were revisited from the baseline 2012 DQA, but there was some turnover (6% [1 of 18] in Kasungu and 32% [5 of 19] in Dowa). HSAs not interviewed in both assessments were excluded (n=6) from the *t* test analysis (sample size for paired *t* test, n=31). Table 2 shows the number of participants in the package, the number selected for the baseline and endline DQA, and the number of respondents with matched data (those interviewed at both baseline and endline).

**TABLE 2.** Sample Size for the DQU Intervention and Evaluation, Dowa and Kasungu Districts, Malawi, 2012–2013

	DQU Implementation	Baseline Assessment	Endline Assessment	Matched Data
Districts	2	2	2	2
Health facilities	69	10	9	9
HSAs	426	38	36	31

Abbreviations: DQU, data quality and use; HSA, Health Surveillance Assistant.

### Data Availability and Quality

Table 3 shows the availability and completeness of the monthly reporting forms at the HSA (Form 1A) and health facility (Form 1B) levels. Availability and completeness of forms at the HSA level was maintained in Kasungu but dropped in Dowa, especially in terms of completeness. A form was considered “complete” only if every section was filled in. In most cases, interviewers found that “incomplete” forms were not missing key data but something minor such as signature. Timeliness (proportion submitted before deadline) was not tracked at the health facilities.

**TABLE 3:** Availability and Completeness of Reporting Forms at the HSA and Health Facility Levels for the Previous Month, Baseline (May 2012) vs. Endline (June 2013)

	Kasungu		Dowa		Total	
	Baseline	Endline	Baseline	Endline	Baseline	Endline
**HSA level**^a^						
Available	93% (25/27)	96% (23/24)	95% (57/60)	80% (37/46)	94% (82/87)	86% (60/70)
Complete	74% (20/27)	79% (19/24)	95% (57/60)	63% (29/46)	89% (77/87)	69% (48/70)
**Health facility level**^b^						
Available	Missing	100% (24/24)	100% (23/23)	44% (11/25)	N/A	71% (35/49)
Complete	Missing	100% (24/24)	95% (22/23)	16% (4/25)	N/A	57% (28/49)

Abbreviation: HSA, Health Surveillance Assistant; iCCM, integrated community case management.

^a^ Denominators represent all iCCM-trained HSAs associated with selected health facilities that would be expected to submit reports.

^b^ Denominators represent all health facilities supporting iCCM that would be expected to submit reports to the district.

At the health facility level, baseline data from Kasungu were not available due to a data collection error but the endline rates show good reporting. Dowa experienced a large drop in availability of forms, reportedly due to lack of blank forms and supplies.

**FIGURE 2:**
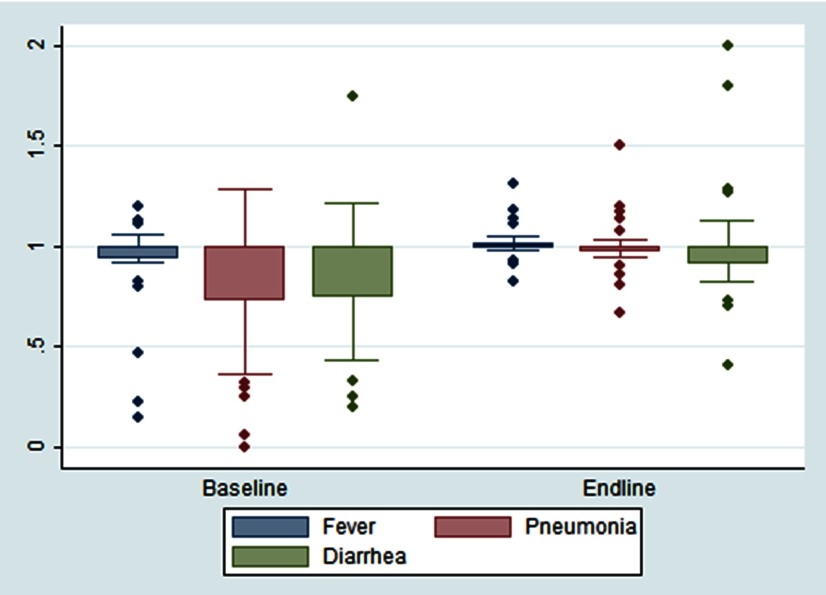
HSA Caseload Reporting Consistency at Baseline (2012) and Endline (2013) for Fever, Diarrhea, and Pneumonia, Dowa and Kasungu Districts Combined, Malawi Abbreviations: HSA, Health Surveillance Assistant; RVR, results verification ratio. An RVR of 1.00 indicates perfect reporting, while less than 1.00 indicates overreporting and greater than 1.00 underreporting.

Figure 2 shows the consistency of routine reporting for child illness at the HSA level at baseline and endline for the most recent reporting month with complete data. Both Dowa and Kasungu showed significant improvements in reporting consistency for fast breathing (i.e., suspected pneumonia cases), from overreporting cases at baseline (RVR=0.82) to no reporting inconsistency at endline (RVR=1.0) (*P*=.02). Other non-significant improvements were measured for fever illness and patient gender.

Both study districts showed significant improvements in reporting consistency for fast breathing.

Changes in reporting consistency were less apparent for drugs dispensed. Reporting quality was maintained for lumefantrine-artemether and cotrimoxazole but decreased for oral rehydration salts. There was also increased variation among the HSAs (Supplement 3).

The sample sizes for health facility consistency of reporting were too small for this analysis. However, at both baseline and endline, the reporting consistency was adequate but with large variation in consistency for certain indicators, particularly in Dowa district (data not shown).

### Data Display and Use

**FIGURE 3:**
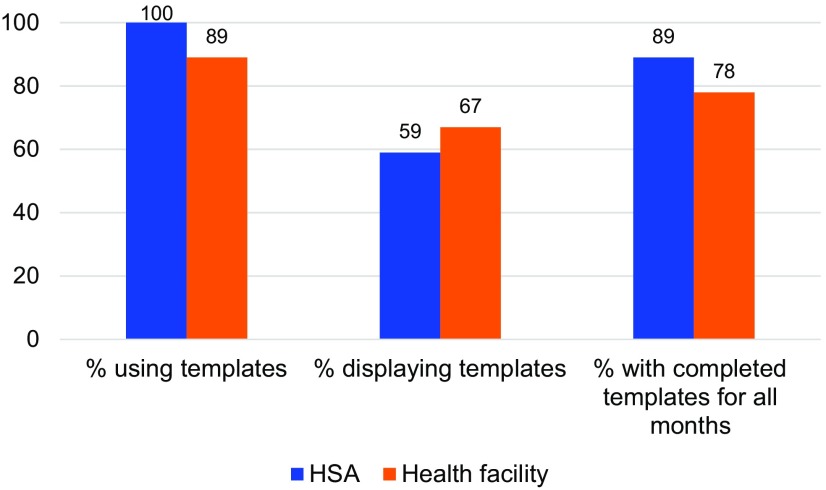
Wall Chart Template Use at the HSA and Health Facility Levels at Endline (2013), Dowa and Kasungu Districts Combined, Malawi Abbreviation: HSA, Health Surveillance Assistant.

Figure 3 shows use of the wall chart template at the HSA and health facility levels at endline. All participants were trained and almost all were using the wall charts. The median time to complete the wall chart for that month was 1 hour. All participants reported that the package training was useful as a job aid and all components would be helpful to scale up in other districts. About half of the HSAs were not displaying the wall charts because their village clinic was not held in a permanent structure. The large majority (90%) of the HSAs and three-quarters of the health facilities had the wall charts completed for every month since January 2013.

Participants were asked whether any program decisions were based on the template data and to give an example. Most HSAs mentioned they used the data from the wall charts to inform their community health education activities. For instance, if HSAs noticed an increase of malaria cases, the HSA would sensitize communities to sleep under mosquito nets.

*Yes, when malaria cases or fever cases were high, the village clinic volunteers and the HSA agreed to conduct health education on malaria and consistent use of treated mosquito nets.* –Dowa HSA as summarized by interviewer

Participants reported that the ability to show an increase in the number of cases resulted in more preventive actions. Several reported holding community talks to discuss increases in child illness cases. Stock-out data were reported to have been used to inform communities that they should seek care at the health facilities in the short-term until the HSA stocks were replenished. HSAs reported using the wall chart data to lobby their supervisors for more drugs.

Most HSAs mentioned they used wall chart data to inform their community health education activities.

In a couple of cases, HSAs asked communities to build permanent structures to house sick-child clinics so they had a place to provide iCCM services and display the wall charts. Participants in this study said that it was very valuable to have the data displayed so that communities could see the information.

*Yes, [the] Village Clinic Committee used the information after getting satisfied with the importance of the templates [wall charts]. The village clinic committee and the community had agreed to contribute money and buy a clinic door [for displaying the wall charts to the community].* –Dowa HSA summarized by interviewer

Senior HSAs at the health facilities reported using their template data to make staffing decisions, e.g., deploying HSAs to vacant areas and asking the district to allocate additional iCCM-trained HSAs. One respondent indicated that the wall charts helped to better track the number of stock-outs and ensure timely reporting by the HSAs.

Another senior HSA reported that an unusually high number of pneumonia cases were reported, so the supervisor convened a meeting and provided refreshers on following the iCCM manual and counting respiratory rates. At the health facilities, participants liked that the data were now available to all, whereas before it was kept by 1 person and not everyone had access to it. The benchmarks and action thresholds were reported to provide helpful guidance.

*It [wall charts with action thresholds] reminds you if you are not doing good to pull up socks and if you are doing good to continue. It is easy to interpret.* –Kasungu senior HSA summarized by interviewer

### Costs

The total implementation cost of this activity was US$11,833 or US$172 per health facility. This included all costs of the TOT, the health facility/HSAs trainings in both districts, supplies and materials, and the 1-week supervisory visit. Costs such as transportation, per diems, refreshments, and printing are included, but staff salaries were not included.

## DISCUSSION

Our results show that provision of wall charts to community and facility health workers to organize and view monthly iCCM reporting data, along with additional training, leads to more data-based decision making. We also found evidence of improvements in reporting consistency but not in availability or completeness of reporting. It is important to note that even at baseline, on average, we found adequate reporting consistency for most indicators despite a few HSAs with very poor reporting. This finding is similar to a study in Mozambique that also found adequate reporting of facility-based records.^20^

Provision of wall charts to community and facility workers to organize and view monthly data leads to more data-based decision making.

In terms of feasibility, the DQU package was well received from the HSAs to the MOH program managers at the national level. The training takes only a half-day per health facility, can be facilitated by district health staff, and requires minimal supplies. Costs will vary based on location, but we consider it an inexpensive investment in the Malawian context. This package could easily be embedded in many community-based health programs.

Our study demonstrated that program support and district management were important mediators of the effectiveness of the DQU package. There was turnover of iCCM supporting agencies and of district staff in the middle of the package implementation, which may have influenced the findings. During the baseline DQA, support from Save the Children for iCCM in Dowa included a district-based project officer who followed up with facilities to obtain complete reports and to resupply iCCM forms, but the project closed in March 2013, prior to the endline DQA. During the baseline DQA, Kasungu received support through WHO and UNICEF, although they did not provide direct staff to assist with reporting. Also, the district had not yet begun supervision and mentoring with the revised checklists during the study period, but by the endline DQA this activity was underway in the sampled health facilities. We found a higher rate of turnover in Dowa district including turnover in the Deputy IMCI coordinator position (personal communication, Humphreys Nsona, Program Manager, IMCI Unit, Ministry of Health, Malawi) found at endline. This data use intervention is promising, but turnover and other issues at the district level may limit the potential effect of any data improvement program and must be continually monitored and addressed.

Program support and district management were important mediators of the effectiveness of the data quality and use intervention.

Since this study, Malawi has scaled up use of the open-source software District Health Information System 2 (DHIS 2)—an electronic and web-based health information system—replacing the previous paper-based system.^21^ The DHIS 2 software integrates the community-based iCCM data with the facility-based information on child illness treatment. We developed district-level Excel-based electronic data display for the DQU package, but it was not used, partly due to the shift to DHIS 2 that was happening concurrently. There is the potential for similar data visualizations to be integrated into the dashboard function of DHIS 2 to automatically generate the graphs based on data entered at the district level. Unfortunately, the system is not available at the health facility level due to computer and Internet connectivity shortages. Health workers at the facility and community levels still require a paper-based system for organizing and visualizing their data.

There have been ongoing efforts on the behalf of partners and the MOH to expand implementation of the DQU package while modifying it to address updates to the iCCM program and other community-based packages implemented by HSAs through the WHO Rapid Access Expansion Programme (RAcE) (Box 2). As of September 2016, the DQU package was being implemented in 15 of Malawi's 29 districts and had been adapted for use in iCCM programs in Mozambique and Nigeria.

BOX 2 **Modifications to the DQU Implementation in 2016**

- Updating the malaria graph to chart rapid diagnostic test positive (RDT+) cases after the introduction of RDTs
- Addition of a graph to capture newborn home visits
- Combining the individual wall charts into a single large poster format for durability and ease of display
- Dropping provision of calculators to reduce the cost of the intervention since most HSAs now have mobile phones with a calculator function

**Figure:**
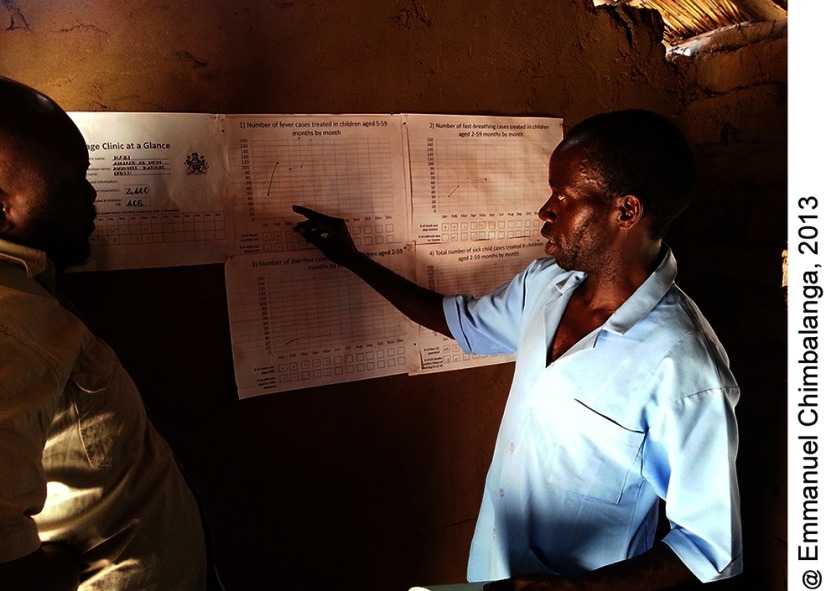
Health Surveillance Assistants in Malawi review iCCM data using wall chart templates.

As of September 2016, the DQU package was being implemented in 15 of Malawi's 29 districts and had been adapted for use in Mozambique and Nigeria.

Unfortunately the scale up of DQU as part of RAcE shows more inconsistent use of the tools. In late 2016, Save the Children conducted a survey of 66 HSAs and only 50% of these had been trained in the package and had received the wall chart templates. Of those trained and who had received the wall chart templates, only 40% had updated data for the last 3 months and only 57% had wall charts displayed (personal communication, T Guenther, Advisor, Save the Children, February 2017). Continued use of the wall charts will require additional investments in supervision and reinforcement. However, if the wall charts are embedded into other community-based programs, supervision and reinforcement could be done during routine supervision or mentoring visits.

There is increased global recognition of the importance of accurate, timely, and available data in improving the health of children in low- and middle-income countries. Sustainable Development Goal 17.18 aims to increase capacity for generating health data.^22^ Limited use and dissemination of data have been identified as a major barrier to effective HMISs.^11^^,^^23^ Since this study, others have documented data use and quality improvement interventions such as enhanced planning and reporting tools in Ghana; electronic database dashboards showing relevant facility, district, and provincial data in Mozambique; electronic patient record systems in Rwanda; and quarterly data use workshops in Tanzania.^24^^,^^25^

### Limitations

This study had important limitations. First, we purposively selected districts close to the capital to reduce transportation costs and these districts are not representative of all of Malawi. More remote districts may have less supervision, training, and access to other health system supports, so the study findings may not be generalizable. Additionally, the period of implementation was at minimum 3 months. It is possible the study findings would be different had we been able to evaluate the intervention after 6 or 12 months of implementation. Furthermore, the HSAs and health facility staff were informed of the endline DQA in advance of the study. It is possible they completed and displayed the wall charts only because they knew there would be a follow-up observation. Finally, examples of data-based decision making were self-reported and we were unable to verify any of the examples given. However, through routine supervision, the staff noted that the wall charts were being filled in on a monthly basis.

## CONCLUSION

Routine data quality is a continual concern for monitoring iCCM programs. If health staff have better access to the data and assistance with interpretation and analysis, monitoring data may be seen as more valuable and the quality more important. This pilot project shows that given the opportunity to access and visualize the data along with supervision support, community and facility health workers can use their data to improve programs at the local level.

## Supplementary Material

Supplement 1

## Notes

**Peer Reviewed**
