# Supplementary material for: Using Data to Improve Programs: Assessment of a Data Quality and Use Intervention Package for Integrated Community Case Management in Malawi
Source: Glob Health Sci Pract. 2017 Sep 27;5(3):355–66. doi: 10.9745/GHSP-D-17-00103 (PMC5620334; doi:10.9745/GHSP-D-17-00103)
Supplement: Supplement 1 [file GHSP-D-17-00103_index.html]

Supplement to Using Data to Improve Programs: Assessment of a Data Quality and Use Intervention Package for Integrated Community Case Management in Malawi | Global Health: Science and Practice

## Supplemental material

- Text s01, PDF - Text s01, PDF
- Text s02, PDF - Text s02, PDF
- Text s03, PDF - Text s03, PDF
