# Supplementary material for: Using Data to Improve Programs: Assessment of a Data Quality and Use Intervention Package for Integrated Community Case Management in Malawi
Source: Glob Health Sci Pract. 2017 Sep 27;5(3):355–66. doi: 10.9745/GHSP-D-17-00103 (PMC5620334; doi:10.9745/GHSP-D-17-00103)

Hazel E, Chimbalanga E, Chimuna T, et al. National assessment of a data quality and use intervention package for integrated community case management in Malawi. *Glob Health Sci Pract*. 2017;5(3).
https://doi.org/10.745/GHSP-D-17-00103

Supplement 1. Wall Chart Templates to Display iCCM Implementation Strength Data at the Health Surveillance Assistant (HSA) Level

Village Clinic at a Glance

Village clinic name: ______________________
HSA name: ______________________
HSA supervisor name: ______________________
Facility name: ______________________

Background information:

Catchment population: [ ]

Estimated # children U5: [ ]

Support and supervision monthly summary (tick if received):

Month:	Jan	Feb	Mar	Apr	May	Jun	July	Aug	Sep	Oct	Nov	Dec
Supervision Visit												
Mentoring												

2) Number of fast breathing cases treated in children aged 2-59 months by month

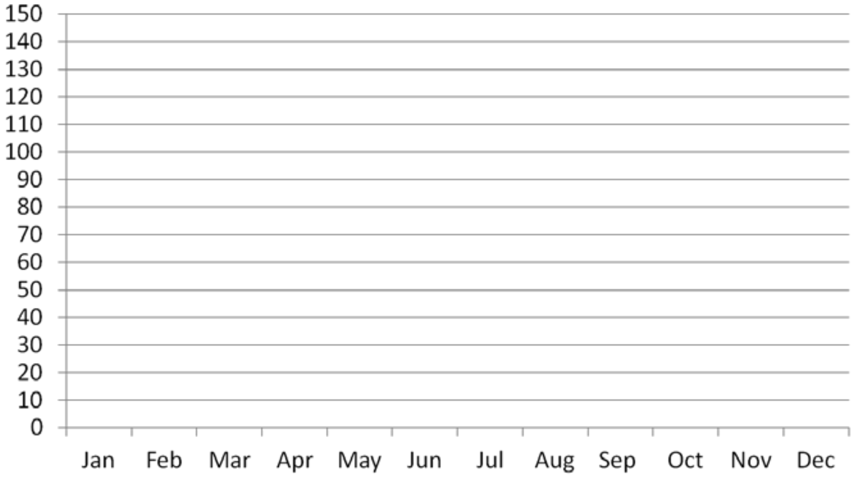

# of stock-out days Cotrim												
# of referrals due to stockout												

Hazel E, Chimbalanga E, Chimuna T, et al. National assessment of a data quality and use intervention package for integrated community case management in Malawi. *Glob Health Sci Pract*. 2017;5(3).
https://doi.org/10.745/GHSP-D-17-00103

1) Number of fever cases treated in children aged 5-59 months by month

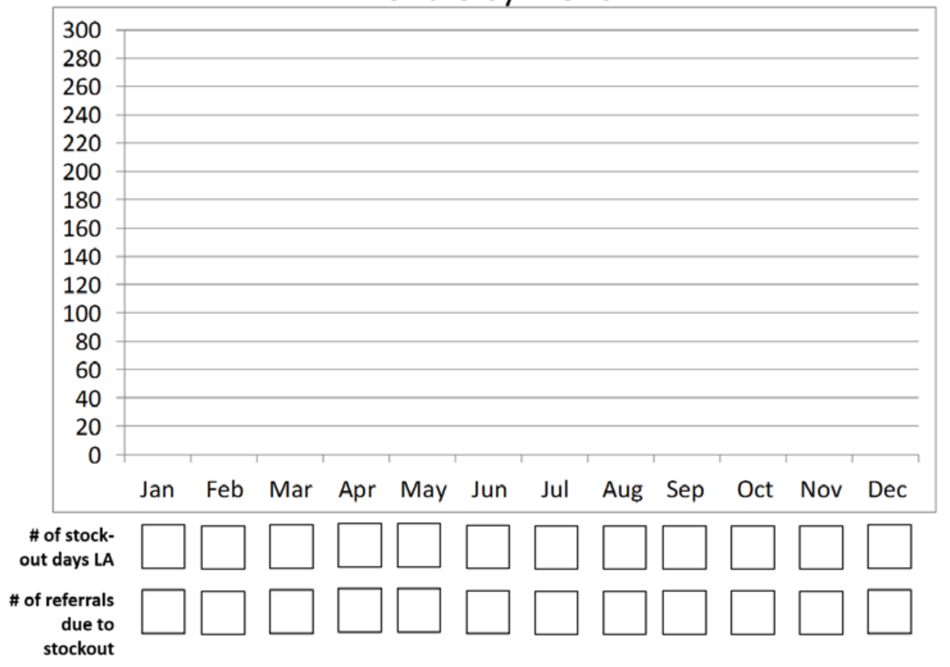

3) Number of diarrhea cases treated in children aged 2-59 months by month

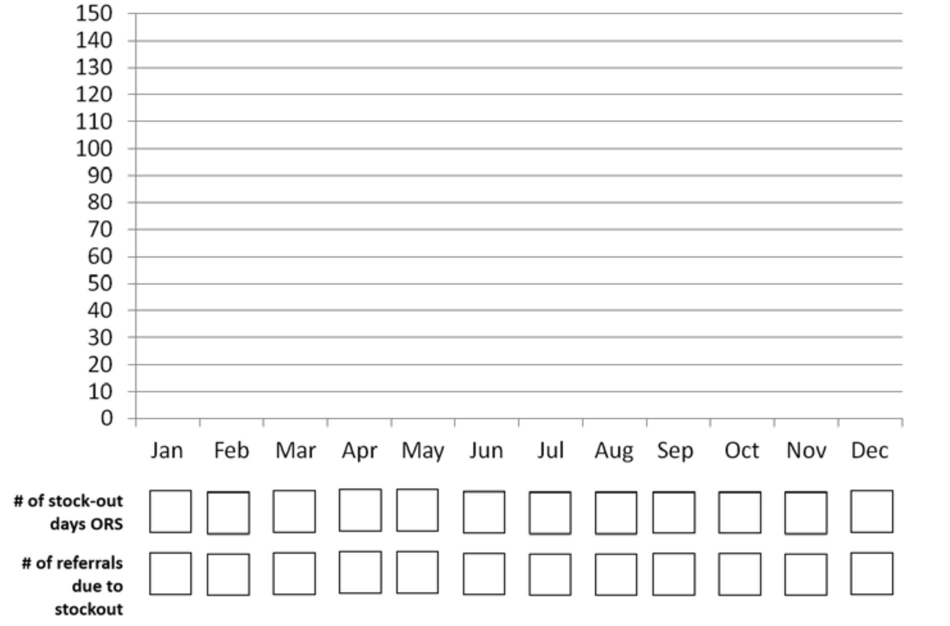

Hazel E, Chimbalanga E, Chimuna T, et al. National assessment of a data quality and use intervention package for integrated community case management in Malawi. *Glob Health Sci Pract*. 2017;5(3).
https://doi.org/10.745/GHSP-D-17-00103

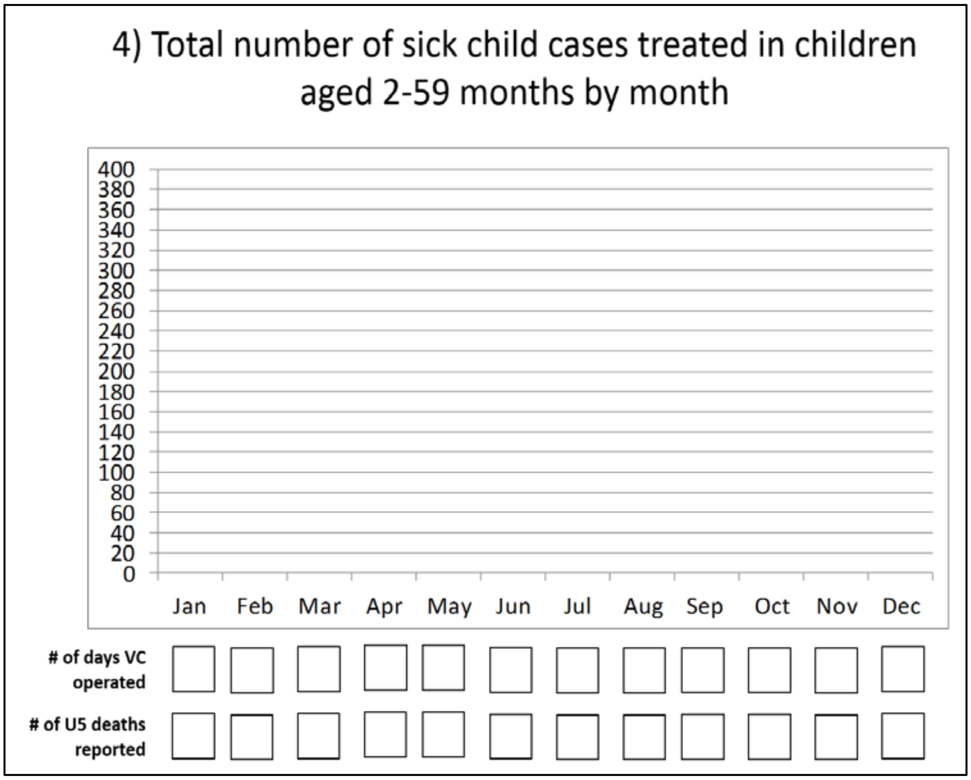

Supplement: Supplement 1 [file 17-00103-Hazel-Supplement1.pdf]
