# Supplementary material for: Using Data to Improve Programs: Assessment of a Data Quality and Use Intervention Package for Integrated Community Case Management in Malawi
Source: Glob Health Sci Pract. 2017 Sep 27;5(3):355–66. doi: 10.9745/GHSP-D-17-00103 (PMC5620334; doi:10.9745/GHSP-D-17-00103)

Hazel E, Chimbalanga E, Chimuna T, et al. National assessment of a data quality and use intervention package for integrated community case management in Malawi. *Glob Health Sci Pract*. 2017;5(3).
https://doi.org/10.745/GHSP-D-17-00103

Supplement 2. Wall Chart Templates to Display iCCM Implementation Strength Data at the Health Facility Level

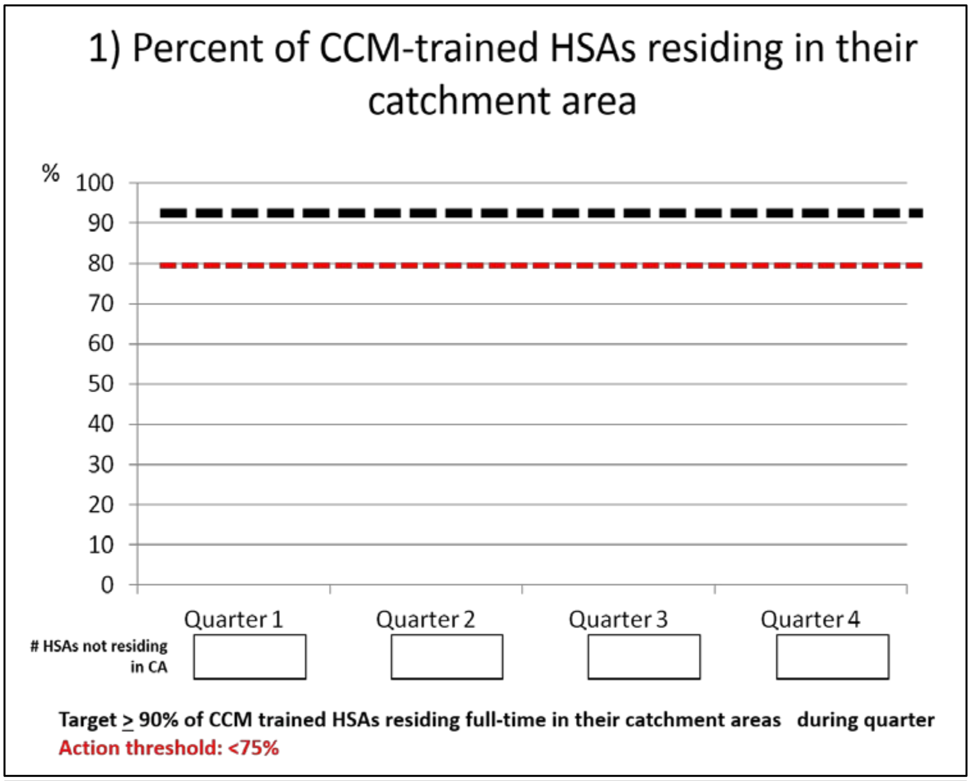

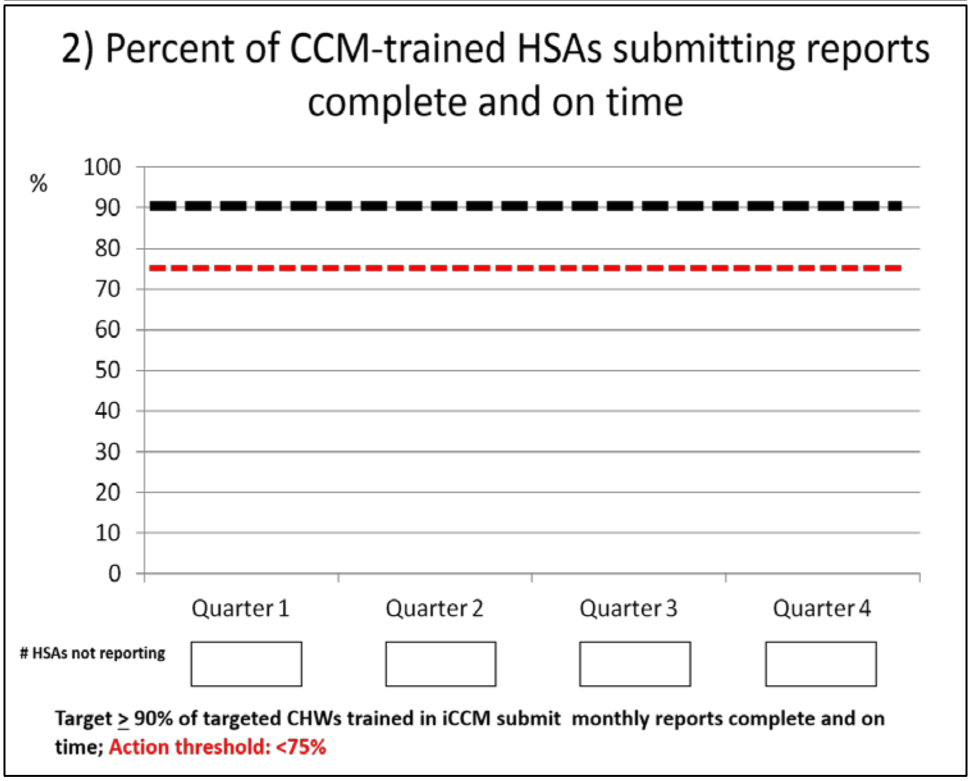

Hazel E, Chimbalanga E, Chimuna T, et al. National assessment of a data quality and use intervention package for integrated community case management in Malawi. *Glob Health Sci Pract*. 2017;5(3). https://doi.org/10.745/GHSP-D-17-00103

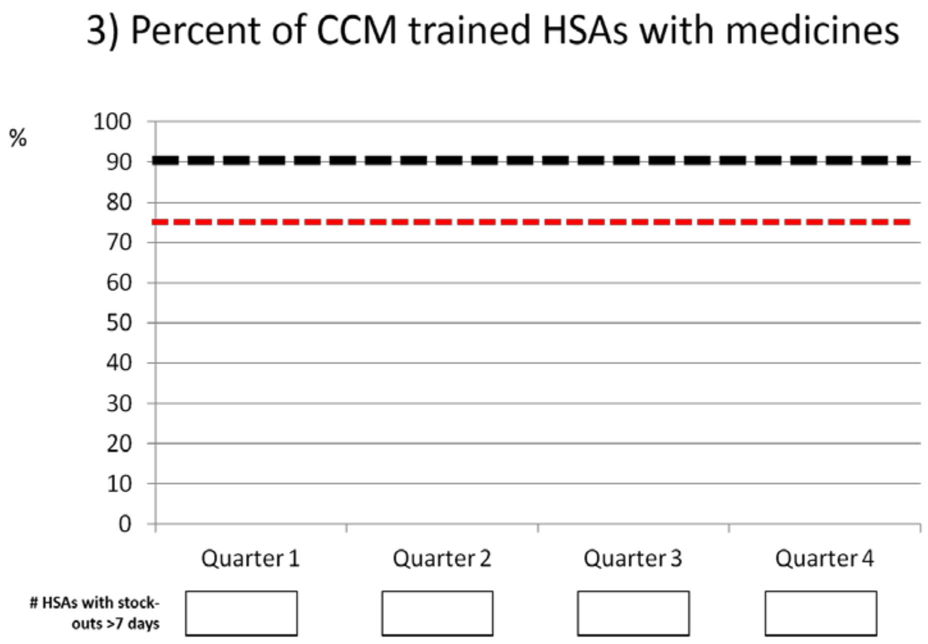

Target ≥ 90% of targeted HSAs trained in iCCM report no stock-outs of LA (or all essential meds) lasting >7 days in quarter; Action threshold: <75%

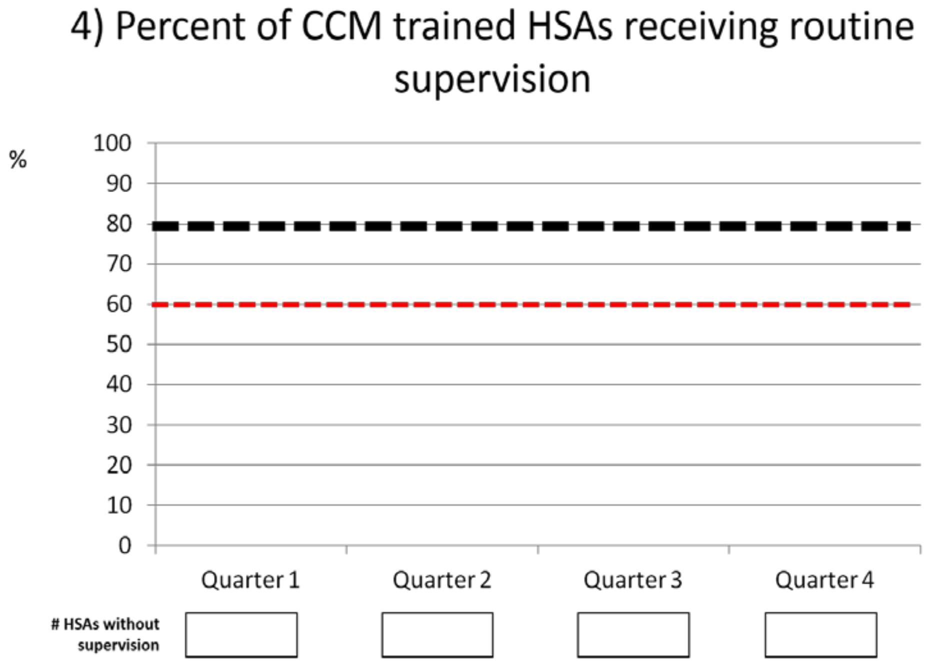

Target ≥ 80% of targeted iCCM-trained HSAs receive ≥ 1 routine supervision/quarter ; Action threshold: <60%

Hazel E, Chimbalanga E, Chimuna T, et al. National assessment of a data quality and use intervention package for integrated community case management in Malawi. *Glob Health Sci Pract*. 2017;5(3).
https://doi.org/10.745/GHSP-D-17-00103

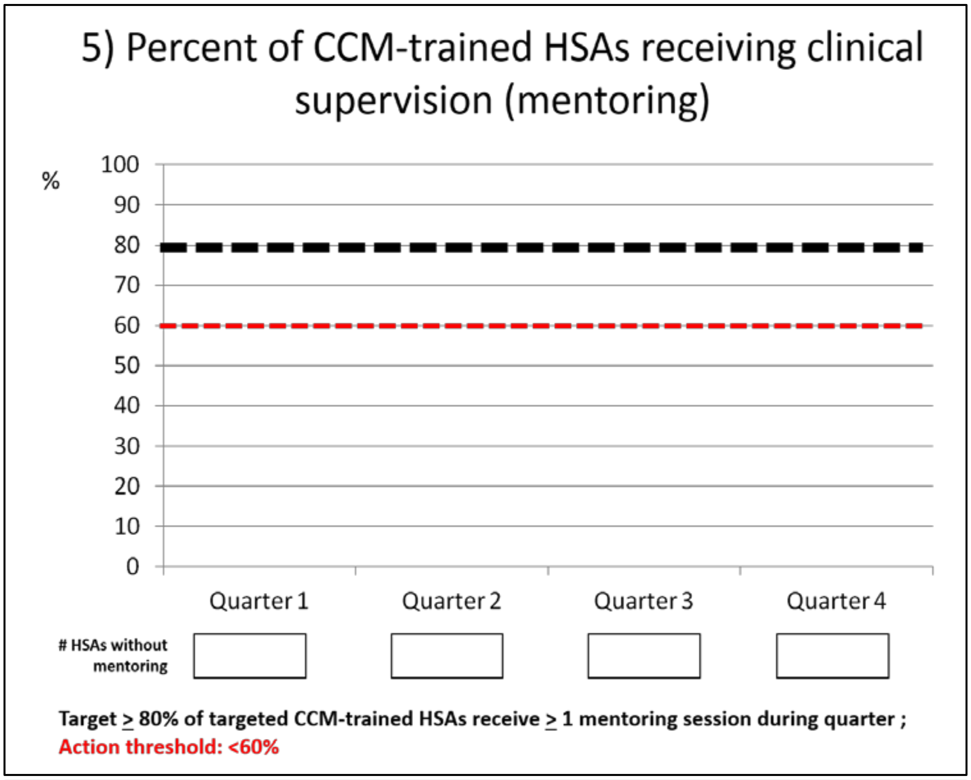

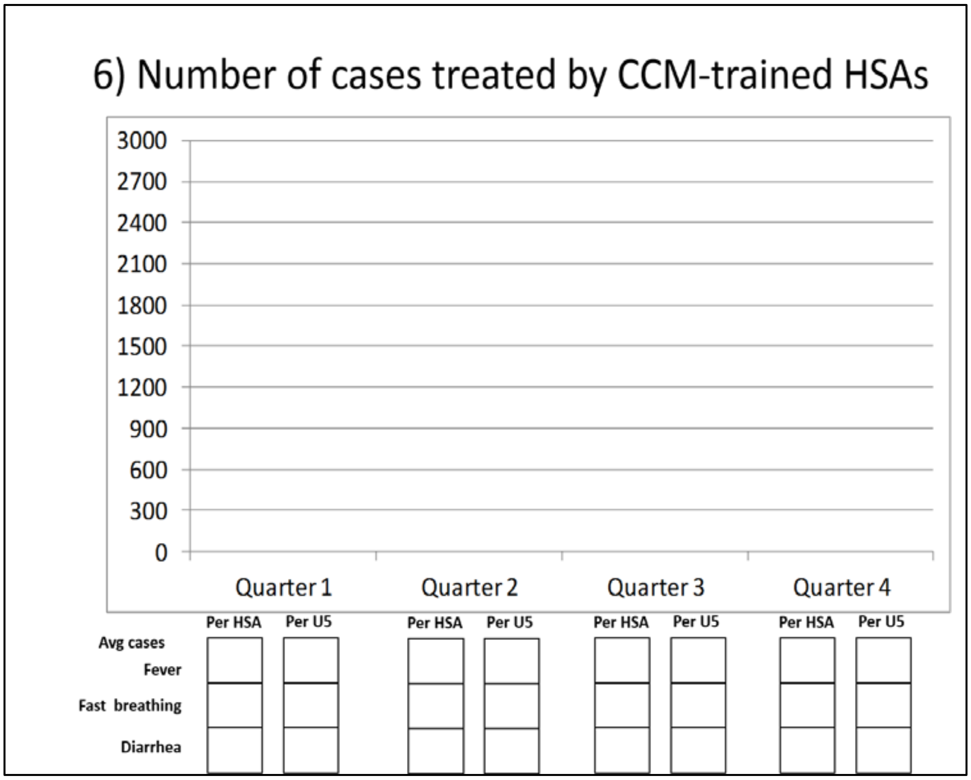

Supplement: Supplement 1 [file 17-00103-Hazel-Supplement2.pdf]
