# Supplementary material for: Using Data to Improve Programs: Assessment of a Data Quality and Use Intervention Package for Integrated Community Case Management in Malawi
Source: Glob Health Sci Pract. 2017 Sep 27;5(3):355–66. doi: 10.9745/GHSP-D-17-00103 (PMC5620334; doi:10.9745/GHSP-D-17-00103)
Supplement: Supplement 1 [file 17-00103-Hazel-Supplement3.pdf]

Hazel E, Chimbalanga E, Chimuna T, et al. National assessment of a data quality and use intervention package for integrated community case management in Malawi. *Glob Health Sci Pract*. 2017;5(3).
https://doi.org/10.745/GHSP-D-17-00103

Supplement 3. Reporting Consistency for HSAs by District and in Total, Malawi, Baseline (2012) vs. Endline (2013)

	**Dowa**		**Kasungu**		**Total**		
	**Baseline (n=20)**	**Endline (n=17)**	**Baseline (n=18)**	**Endline (n=19)**	**Baseline (n=38)**	**Endline (n=36)**	***P* Value[a]**
Fever cases	0.91 (0.07)	1.01 (0.02)	0.91 (0.08)	1.02 (0.02)	0.91 (0.05)	1.01 (0.01)	.07
Diarrhea cases	0.87 (0.06)	1.04 (0.09)	0.95 (0.08)	1.00 (0.02)	0.91 (0.05)	1.02 (0.05)	.50
Fast breathing cases	0.92 (0.04)	1.02 (0.04)	0.69 (0.10)	0.99 (0.02)	0.82 (0.05)	1.00 (0.02)	.02
No. of male cases	0.97 (0.04)	0.99 (0.04)	0.81 (0.08)	1.02 (0.02)	0.91 (0.04)	1.00 (0.02)	.10
No. of female cases	1.06 (0.07)	0.99 (0.03)	0.81 (0.08)	1.00 (0.01)	0.95 (0.06)	0.99 (0.02)	.75
**Stocks distributed**							
Lumefantrine-artemether	0.95 (0.04)	1.14 (0.09)	1.12 (0.08)	1.01 (0.02)	1.03 (0.04)	1.08 (0.05)	.70
Cotrimoxazole	0.89 (0.06)	1.02 (0.05)	0.97 (0.05)	0.87 (0.08)	0.93 (0.04)	0.95 (0.05)	.69
Oral rehydration salts	0.89 (0.06)	1.82 (0.76)	0.91 (0.10)	1.03 (0.12)	0.92 (0.05)	1.46 (0.42)	.20

Abbreviations: HSA, Health Surveillance Assistant; RVR, results verification ratio; SE, standard error.

All data reported as RVR (SE). An RVR of 1.00 is perfect reporting, while less than 1.00 indicates overreporting and greater than 1.00 underreporting.

[a] Paired *t* test analysis was used. HSAs not interviewed at endline were excluded (n=7) from the *t* test analysis (sample size for paired *t* test, n=31).
